# Association Between Vitamin D Deficiency and Subclinical Cardiovascular Symptoms Among Young Adults in Pakistan

**DOI:** 10.7759/cureus.89134

**Published:** 2025-07-31

**Authors:** Sandhya Sahani, Adu Yiadom Boakye, Maherullah Kasi, Hafsa Ahmed, Naveen Shaikh, Ayesha Mansoor, Syed Saif Ali, Faheem Ashraf, Bushra Kaynat, Muhammad Imran Sharif, Haider Alam

**Affiliations:** 1 Emergency Medicine, Tomo Riba Institute of Health and Medical Sciences, Naharlagun, IND; 2 Medicine, Zaporizhzhia State Medical University, Zaporizhzhia, UKR; 3 Cardiology, Ternopil National Medical University, Ternopil, UKR; 4 General Surgery, Sandeman Provincial Hospital Quetta, Quetta, PAK; 5 Internal Medicine, Mayo Hospital Lahore, Lahore, PAK; 6 Epidemiology and Biostatistics, Aga Khan University, Karachi, PAK; 7 General Practice, Response Plus Medical Services LLC, Abu Dhabi, ARE; 8 Special Pathology, Community Medicine, and ENT, Allied Hospital, Faisalabad Medical University, Faisalabad, PAK; 9 Medicine, Karachi Medical and Dental College, Karachi, PAK; 10 Medicine, Quaid-e-Azam Medical College, Lahore, PAK; 11 Anaesthesia, Allama Iqbal Teaching Hospital Dera Ghazi Khan, Multan, PAK; 12 General Medicine, Almazroui Medical Center, Abu Dhabi, ARE; 13 Medicine, MedNova Research Institute, Islamabad, PAK; 14 Medicine, Shifa College of Medicine, Shifa Tameer-E-Millat University, Islamabad, PAK

**Keywords:** pakistan, physical activity, subclinical cardiovascular symptoms, vitamin d deficiency, young adults

## Abstract

Background

Vitamin D is an essential component of human health, and its effects may be felt outside of musculoskeletal development. Vitamin D deficiency is common among many individuals, including those with adequate sunlight exposure. However, evidence linking vitamin D deficiency to subclinical cardiovascular symptoms (such as mild chest discomfort, palpitations, exertional breathlessness, or fatigue) in otherwise healthy young adults is limited. The study aimed to investigate the relationship between subclinical cardiovascular symptoms and vitamin D deficiency, as well as the influence of demographic and lifestyle factors, in young adults in Pakistan.

Methods

The research involved a cross-sectional investigation between November 2024 and May 2025 across community centres, outpatient facilities, and universities in both urban and rural areas of Pakistan. Convenience sampling was employed to recruit 402 participants aged 18 to 35 for the study. The data were collected using structured questionnaires that included a demographic profile, the Vitamin D Deficiency Risk Assessment Questionnaire (VDRAQ), and the Duke Activity Status Index (DASI). VDRAQ was used to assess vitamin D deficiency risk based on lifestyle and demographic factors, while DASI was used to evaluate subclinical cardiovascular symptoms by measuring physical functioning. The dependent variable was the vitamin D status, and its relationship with subclinical cardiovascular indicators was determined using descriptive statistics, t-tests, ANOVA, Pearson correlation, and linear regression via the IBM SPSS 26 (IBM Corp., Armonk, USA).

Results

Out of 402 participants, 51% were men and 49% were women, with the most significant percentage falling in the age bracket of 24-26 years. There was a significant negative correlation between VDRAQ and DASI (r = -0.271, p < 0.001), indicating that a high risk of vitamin D deficiency was associated with poorer physical functioning. The VDRAQ scores were greater and the DASI scores were lower among females as compared to males (p = 0.050 and p = 0.040, respectively). The regression result ensured that VDRAQ scores were significant in predicting DASI scores (beta = -0.271, p < 0.001). Vitamin D risk levels were also significantly linked to marital status and age.

Conclusion

This research is crucial in establishing an essential relationship between the risk of vitamin D deficiency and a decline in physical activities, representing a marker of cardiovascular dysfunction at its earliest stages in a group of young adults in Pakistan. These results underscore the importance of screening, raising societal awareness, and promoting behavioural changes to help young people improve their vitamin D status and reduce their long-term cardiovascular risk.

## Introduction

Vitamin D deficiency is a primary global health concern, affecting nearly one billion individuals worldwide [1]. While it is classically linked with musculoskeletal complications, an increasing body of evidence points toward its significant role in cardiovascular health [2]. Vitamin D receptors are present in vascular smooth muscle, endothelium, and cardiomyocytes, suggesting that its deficiency may adversely affect cardiovascular function through mechanisms such as endothelial dysfunction, inflammation, and increased arterial stiffness [3]. Vitamin D regulates nitric oxide (NO) synthesis in endothelial cells, playing a protective role against endothelial dysfunction (ED), a precursor to atherosclerosis. It counteracts oxidative stress and inflammation by inhibiting the production of reactive oxygen species (ROS) and suppressing pro-inflammatory mediators, such as Tumor Necrosis Factor-alpha (TNF-α) and Interleukin-6 (IL-6), thereby improving endothelial function and reducing cardiovascular risk [4].

Globally, approximately 40% of adults have suboptimal vitamin D levels, with higher prevalence in Middle Eastern and South Asian countries due to limited sun exposure, darker skin pigmentation, and cultural clothing norms [5]. In Pakistan, despite abundant sunlight, studies show that between 60-80% of the population is vitamin D deficient [6]. Young adults aged 18 to 26 years are at increased risk of vitamin D insufficiency due to inadequate milk intake and concerns about sun exposure, particularly from the fear of developing skin cancer. These lifestyle factors contribute significantly to the prevalence of vitamin D insufficiency in this age group [7]. Compared to Western countries like the United States (24-32%) and Canada (37%), Pakistan's burden is significantly higher [8]. Despite ample sunshine in Pakistan, approximately 53.5% of the population is vitamin D-deficient, with only 15.3% having normal levels. Additionally, around 64.6% of healthy individuals in Pakistan have vitamin D levels below 30 ng/mL, indicating widespread deficiency across various demographics [9,10].

Parallel to this, cardiovascular disease remains the leading cause of mortality worldwide. While much focus has been on overt cardiac conditions, subclinical cardiovascular symptoms - such as mild chest discomfort, unexplained fatigue, exertional breathlessness, palpitations, and occasional dizziness - may represent early manifestations of vascular dysfunction [11]. These symptoms often precede diagnosable heart disease and are increasingly reported among young adults, particularly those exposed to emerging metabolic and inflammatory risk factors [12].

Vitamin D deficiency may be an under-recognized contributor to these subclinical symptoms. Studies have linked low serum 25(OH)D levels (<20 ng/mL) with early markers of cardiovascular compromise, including increased carotid intima-media thickness, impaired flow-mediated dilation, and systemic inflammation [13]. However, there is a lack of research specifically assessing this association in apparently healthy young adults from high-risk regions like Pakistan.

This study aims to investigate the relationship between vitamin D deficiency and subclinical cardiovascular symptoms in young adults in Pakistan. By focusing on an underrepresented population experiencing early cardiac signs without diagnosed disease, this research may aid in early detection and risk-stratification efforts in preventive cardiology.

Rationale

Most young adults are not expected to have a high risk of cardiovascular issues. However, sometimes, despite the lack of an identified heart disease, they may complain about the presence of those problems (including palpitations, shortness of breath, or even an unexplained feeling of tiredness). This is characterized by a series of symptoms that can be interpreted as subclinical signs related to cardiovascular change. Meanwhile, there are several young adults with very low vitamin D, which may have a role to play in the regulation of basic physiology, such as in situations involving the heart and circulation. This age group (18-35 years) is chosen because it represents a critical period in life when early cardiovascular risk factors can develop but may not yet be clinically diagnosed. As young adults begin to establish lifestyle habits such as physical activity, diet, and sun exposure, they may be at a higher risk for early signs of cardiovascular dysfunction. These early signs can contribute to long-term cardiovascular disease risk if not addressed, making this age group a critical focus for preventive cardiology.

In this study, vitamin D deficiency is assessed using the Vitamin D Deficiency Risk Assessment Questionnaire (VDRAQ), which evaluates risk factors such as sun exposure, physical activity, and diet. While serum 25(OH)D levels are the gold standard for diagnosing vitamin D deficiency, the VDRAQ offers a non-invasive and population-friendly approach. A higher score on the VDRAQ correlates with a greater risk of vitamin D deficiency, and individuals at the highest risk, as determined by the questionnaire, are considered to have very low vitamin D levels. This approach allows for practical, large-scale screening without the need for clinical testing. 

This research is aimed at examining the relationship between insufficiency of vitamin D and the occurrence of subclinical cardiovascular manifestations among young adults. Being overlooked during daily clinical practice, the discovery of any correlation between those two conditions might contribute to the spread of awareness and early intervention. In the case of finding a relationship, this may support simple interventions like lifestyle changes or early screening to enhance well-being and further reduce health hazards in the youth.

Objectives

The primary objective of this research is to investigate the relationship between vitamin D deficiency and subclinical cardiovascular symptoms in young adults. Another purpose of the study is to establish the prevalence of vitamin D deficiency in this population and to identify what subclinical cardiovascular manifestations, including palpitations, fatigue, or mild shortness of breath, are most often reported by individuals at high risk of vitamin D deficiency (as assessed by the VDRAQ). The study will also attempt to compare the frequency and severity of these symptoms among people with adequate and inadequate vitamin D levels, as assessed by the VDRAQ. Another aim is to assess gender-based variation in vitamin D status and the manifestation of cardiovascular symptoms in young adults.

## Materials and methods

Study design and methods

The study employed a cross-sectional design to investigate the relationship between vitamin D deficiency and mild cardiovascular symptoms among young adults. Although a cross-sectional design does not enable the determination of a causal relationship, it can play a role in investigating the relationship between vitamin D deficiency and subclinical cardiovascular symptoms in young adults. The design provides meaningful baseline information that can serve as input for future studies examining the causal associations between vitamin D deficiency and cardiovascular health. The research participants were selected from universities, community settings, and outpatient centres, whether private or publicly operated. This sampling approach guaranteed the diversity of the sample and a variety of socioeconomic and educational backgrounds. Young adults between 18 and 26 years of age were eligible to participate in the study [11], provided they had no significant medical history and were not diagnosed with cardiovascular disease.

The information about the participants was gathered using structured questionnaires that included demographic data and two standardised measures. These measures determined the probability of vitamin D deficiency based on lifestyle and health-related parameters and assessed the level of physical functioning and activity. Subclinical cardiovascular symptoms such as fatigue, palpitations, or shortness of breath were captured as part of the responses to the VDRAQ tool, which includes self-reported health indicators associated with vitamin D deficiency. The self-administration and assisted administration methods were employed to complete the questionnaires, with the assistance of skilled interviewers who tailored the approach to the participants' preferences and comprehension levels. All participants provided informed consent before the collection of data.

The approach enabled the study to examine the precursors of cardiovascular stress regarding vitamin D status and physical activity patterns in a young adult population. The results can help inform the development of preventive measures for cardiovascular and metabolic health in these age groups.

Sample size and technique

The study was considered to have an infinite population, as no information was available on the prevalence of young adults within the general population who are susceptible to vitamin D deficiency or exhibit subclinical signs of cardiovascular disease. The following formula calculates the number of the required sample:

\[n = \frac{Z^2 \cdot p (1 - p)}{d^2}\]

Whereas Z in this formula refers to the standard score that matches the required level of confidence, p is the estimated proportion, and d is the pardonable margin of error. A standard level of trust (95%) was applied (Z = 1.96), and the d value was determined at a significance level of 0.05. The likelihood of a false-negative (p) was set to 0.50 to give the largest possible sample size required [14].

The sample size used in the study was determined based on specific parameters, resulting in a total of 384 participants. However, to ensure that the survey is conducted efficiently and that no data is lost or overlooked, 402 participants were enrolled in the study, with their answers being captured effectively and tabulated accurately. Convenience sampling was used to recruit participants from universities, community centers, and outpatient clinics. The approach enabled the inclusion of available individuals, fulfilled the eligibility requirements, and accepted participation during data collection.

The inclusion and exclusion criteria used by the researcher to select participants are summarised in Table 1.

**Table 1:** Inclusion and Exclusion Criteria for Study Participants

Inclusion Criteria	Exclusion Criteria
Adults aged 18 to 26 years were eligible to participate in the study. This age range was chosen to pinpoint an important stage of young adulthood when lifestyle habits, including sun exposure and physical activity, could be formed. These behaviours can impact either vitamin D deficiency and cardiovascular wellbeing, and that is why such groups would be of special interest in studying early stages of cardiovascular dysfunction [15].	Patients with diagnosed cardiovascular disease.
Being willing to take part and giving informed consent.	Those who use vitamin D supplements regularly.
Being able to comprehend and fill out the questionnaire (with or without help).	Pregnant or lactating women.
No medical history of vitamin D deficiency diagnosed by laboratory measures.	Individuals who have chronic conditions (e.g., renal, hepatic, or endocrine disorders) that could interfere with the vitamin D levels or cardiovascular symptoms.
-	Individuals with a prior diagnosis of vitamin D deficiency were excluded to control for potential confounding factors related to prior treatments or interventions, allowing for a more accurate assessment of subclinical symptoms in individuals without a known history of deficiency.

Data collection tools

This study developed a structured questionnaire divided into three principal sections: demographic information, risk assessment of vitamin D deficiency, and assessment of physical activity and symptoms related to the cardiovascular system. It included questions that were standardised and also created by the researchers themselves to ensure the coverage and relevance of these data.

Demographic information

The initial part collected demographic information to investigate potential relationships between participant characteristics and subclinical cardiovascular symptoms. Such variables include age, gender, marital status, level of education, occupation, and lifestyle, specifically whether they smoke or engage in physical activity. This part allowed studying the characteristics of the samples and making comparisons between subgroups.

Duke Activity Status Index (DASI)

To measure physical functioning and cardiovascular fitness, Hlatky et al. developed the Duke Activity Status Index (DASI) in 1989, which was used to assess participants in the current study. It is a self-administered instrument comprising 12 items that measure an individual's capacity to perform daily activities, such as walking, climbing stairs, doing housework, and engaging in recreational activities. Each activity is assigned a weight proportional to its metabolic equivalent (MET), and the total score (0 to 58.2) is calculated by summing the scores corresponding to activities that the participant indicates they can achieve. Higher scores indicate the best functional capacity. DASI is characterised by high simplicity and efficacy, having been primarily employed in both clinical and research applications. It has demonstrated good internal consistency, with Cronbach's alpha values ranging between 0.70 and 0.86 [16]. The original authors granted permission for the use of the scale.

Vitamin D deficiency risk assessment

The study used a Vitamin D Deficiency Risk Assessment Questionnaire (VDRAQ), developed by Alzahrani and Asghar (2025) and published in *PeerJ Computer Science*, to measure the risk of the participants being diagnosed with vitamin D deficiency [17]. This self-help tool assesses demographic, medication, and lifestyle risk factors such as exposure to the sun, intake, and sunscreen, physical activity, body mass index, and symptoms such as fatigue or pain in bones. Vitamin D status was inferred using the VDRAQ, which evaluates lifestyle factors rather than directly measuring serum 25(OH)D levels. While the VDRAQ includes self-reported symptoms such as fatigue, these symptoms were assessed subjectively by the participants, with no further categorisation or clinical assessment of mild cardiovascular symptoms (such as palpitations or shortness of breath).

A biochemical test of serum 25(OH)D concentration would be more appropriate for measuring vitamin D status, but because the VDRAQ is more practical and non-invasive, the test is viable for conducting in large-scale studies. All the responses will have a definite score depending on their relation to the vitamin D deficiency, and the higher the overall score, the higher the risk. Internal consistencies (Cronbach's alpha = 0.76) and diagnostic accuracy (ROC = 0.78) of the questionnaire were acceptable and reasonable, respectively. Since it is available with the Creative Commons Attribution License (CC BY 4.0), special permission was not needed to use it as long as it was cited properly. It is a non-invasive tool that can be administered simply; it can be used in clinical and population-based research [17].

Table 2 provides a comparative description of the Duke Activity Status Index (DASI) and the Vitamin D Deficiency Risk Assessment Questionnaire (VDRAQ), including their respective strengths, intended uses, areas of coverage, and psychometric properties.

**Table 2:** Overview of Psychometric and Clinical Characteristics of Selected Assessment Tools

Feature	Duke Activity Status Index (DASI)	Vitamin D Deficiency Risk Assessment Questionnaire (VDRAQ)
Developer	Hlatky et al., 1989 [16]	Alzahrani and Asghar, 2025 [17]
Purpose	To assess physical functioning and cardiovascular fitness	To measure the risk of vitamin D deficiency
Type	Self-administered questionnaire	Self-help questionnaire
No. of Items	12	Multiple items (exact number varies)
Assessed Domains	Ability to perform daily and recreational activities (e.g., walking, climbing stairs, housework)	Sun exposure, dietary intake, sunscreen use, physical activity, BMI, and symptoms (e.g., fatigue, bone pain)
Interpretation	The higher the score, the better the functional capacity	Higher scores are associated with an increased likelihood of vitamin D deficiency
Reliability	Cronbach’s alpha: 0.70-0.86	Cronbach’s alpha: 0.76
Application	Clinical and research	Clinical and population-level studies

Procedure

Participants were recruited by inviting them to participate in the research after attaining informed consent on university campuses, in community places, and in outpatient clinics. The data collection process began in November 2024 and concluded in May 2025, spanning a total of five months. Depending on their preferences and level of understanding, participants were allowed to complete the questionnaire either independently or with the assistance of trained research team members. Personal data was not associated with the responses to guarantee the privacy of the answers; all the responses were anonymous. The data collection involved self-reporting by participants of lifestyle factors (e.g., sun exposure, diet, and physical activity) using the Vitamin D Deficiency Risk Assessment Questionnaire (VDRAQ). It is important to note that this study did not directly assess subclinical cardiovascular symptoms such as fatigue or palpitations. Instead, the focus was on lifestyle and health-related factors that might indicate a higher risk for vitamin D deficiency, which has been linked to potential early signs of cardiovascular dysfunction. The use of this procedure enabled the inclusion of young adults from diverse educational and social backgrounds, making the obtained data representative, unbiased, and ethically handled.

Statistical analysis

The analysis of the data was performed in IBM SPSS Statistics version 26 (IBM Corp., Armonk, USA). The demographics of the participants were described using descriptive statistics such as means, standard deviations, frequencies, and percentages. Kolmogorov-Smirnov and Shapiro-Wilk tests were used to test the normalcy of the data. The Kolmogorov-Smirnov test is suitable for larger sample sizes, while the Shapiro-Wilk test is more sensitive for smaller datasets. Both tests were used to ensure that the data met the assumptions of normality required for subsequent statistical analyses. The analysis of correlation by Pearson was used to study the degree of connection between scores of the Vitamin D Deficiency Risk Assessment Questionnaire (VDRAQ) and the Duke Activity Status Index (DASI). Independent t-tests were employed to distinguish the disparities between genders and adherence to strict diets among the individuals in the VDRAQ and DASI scores. One-way ANOVA was used to measure modifications in scores according to the levels of physical activities and marital status. Linear regression analysis was conducted to examine the relationship between VDRAQ scores and DASI scores. The regression model was adjusted for potential confounders, including age, gender, physical activity, and diet, to isolate the effect of vitamin D deficiency risk on physical activity. This adjustment ensured that these factors did not influence the observed relationship between VDRAQ scores and DASI scores.

In this study, multicollinearity was not assessed because the regression model included only one independent variable (VDRAQ score). Multicollinearity is a concern when multiple predictors are included in a regression model and are highly correlated with each other; however, this was not the case here. Therefore, multicollinearity does not apply in this analysis. The impact of age and marital status on the correlation between physical activity and stress was examined using chi-square tests. All statistical tests were conducted at a significance level of p < 0.05 to determine factors that increase the risk of Vitamin D deficiency and low levels of physical activity.

Ethical considerations

The research adhered to all ethical considerations for conducting research with human subjects, conforming to established standards and guidelines. The Institutional Review Board (IRB) of the MedNova Research Institute in Islamabad approved the study protocol before the commencement of data collection (approval no. IRB-2024-0074). This approval of ethics demonstrated that the research met the following principles: respect for persons, protection of welfare, and confidentiality. The participants were adequately informed about the research's intention, the methodologies involved, the risks associated with it, and the potential benefits that might arise. Before participation, written informed consent was obtained from all individuals. Participating in the study was entirely voluntary, and volunteers could withdraw at any point without repercussions. All personal data was confidential and used solely for academic and research purposes, as it was necessary to maintain the privacy of individuals.

Missing data was managed by examining the incomplete responses vigilantly. Where participants did not respond in non-essential parts, their records were not discarded but included in the analysis through the pair-wise deletion method, which helped analyse as much information as possible. Nevertheless, participants who did not complete more than 20% of the survey fields were excluded from the final dataset to maintain the validity and credibility of the findings.

## Results

Table 3 shows the demographic variables of the participants (N = 402). The majority of participants were aged 24-26 years (n = 203, 50.5%), followed by those aged 21-23 years (n = 158, 39.3%), and then those aged 18-20 years (n = 41, 10.2%). An almost equal number was divided based on gender, with a slight predominance of males (n = 203, 50.5%) compared to females (n = 199, 49.5%). In marital status, the most populous respondents were divorced (n = 150, 37.3%), followed by single (n = 147, 36.6%), people who preferred not to disclose (n = 54, 13.4%), and those who were married (n = 51, 12.7%). Education-wise, a large proportion of them were currently pursuing a Bachelor's degree (n = 146, 36.3%), had finished their Bachelor's (n = 120, 29.9%), stopped or interrupted schooling (n = 55, 13.7%), a Master degree (n = 52, 12.9%), or intermediate/A-level education (n = 29, 7.2%). Most participants resided in semi-urban (n = 216, 53.7%), rural (n = 107, 26.6%), or urban (n = 79, 19.7%) areas. In terms of employment, the majority of them were full- or part-time workers (n = 137, 34.1% or, correspondingly, n = 99, 24.6%), others were unemployed (n = 71, 17.7%), intern/trainee (n = 42, 10.4%), full-time students (n = 27, 6.7%, or self-employed/freelancer (n = 26, 6.5%).

**Table 3:** Demographic Characteristics of Participants (N=402) Note. f=frequency, %=percentage

Variable	f	%
Age	-	-
18-20 years	41	10
21-23 years	158	39
24-26 years	203	51
Gender	-	-
Male	203	51
Female	199	49
Marital status	-	-
Single	147	36
Married	51	13
Divorced	150	37
Prefer not to say	54	13
Educational level	-	-
Enrolled in an intermediate/A-level program	29	7
Currently pursuing a Bachelor's degree	146	36
Completed a Bachelor's degree	120	30
Currently pursuing a Master's degree	52	13
Dropped out/paused education	55	14
Residential area	-	-
Urban	79	20
Semi-urban	216	54
Rural	107	26
Employment status	-	-
Full-time student	27	7
Part-time student + part-time work	99	24
Employed full-time	137	34
Unemployed	71	18
Intern/trainee	42	10
Freelancing/self-employed	26	6.5

**Figure 1:**
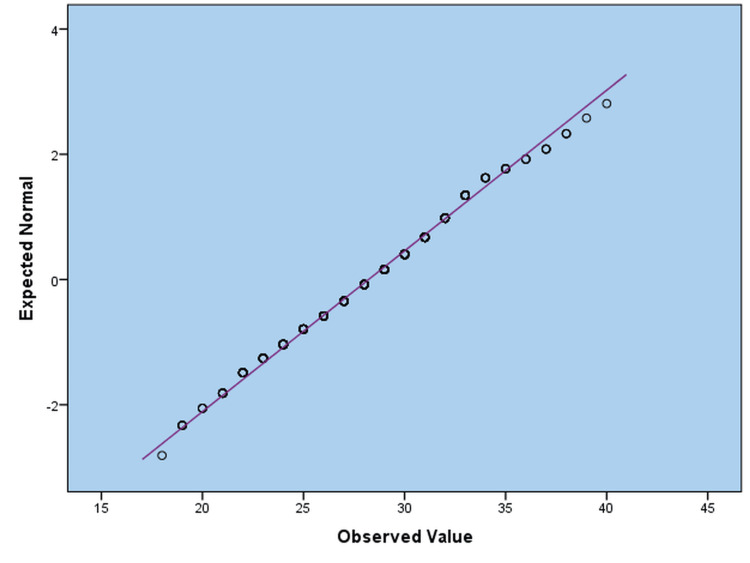
Standard Q-Q Plot Assessing the Distribution of Vitamin D Deficiency Risk Assessment Questionnaire (VDRAQ) Scores Note. The plot shows that the observed values closely follow the expected regular distribution line, indicating that the variable vitamin D deficiency risk assessment questionnaire approximates normality. Based on VDRAQ [17], which assesses vitamin D deficiency risk based on lifestyle and demographic factors.

Figure 1 illustrates the Q-Q verification of the VDRAQ, which evaluates the scores of the VDRAQ. The sample points on the plot correlate well with the supposed standard distribution curve, indicating that the VDRAQ scores are close to a normal distribution. This indicates that the measurable variable represented in the questionnaire is usually distributed.

**Figure 2:**
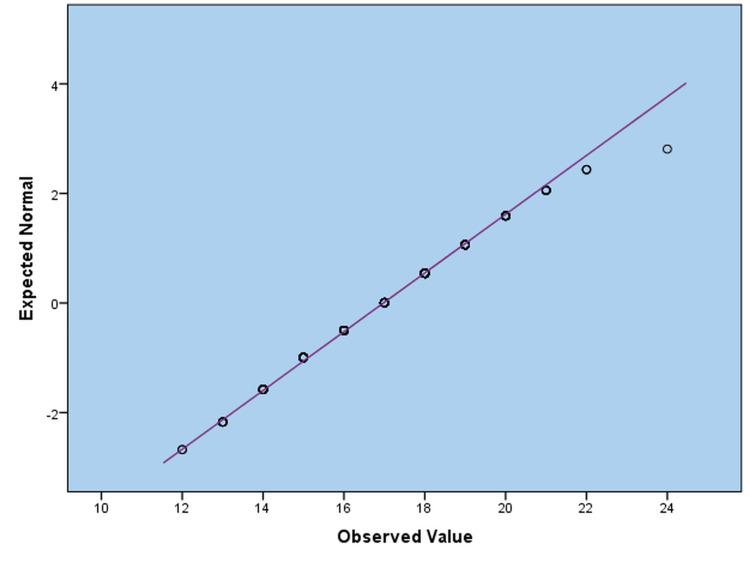
Standard Q-Q Plot Assessing the Distribution of Duke Activity Status Index (DASI) Scores Note. The data points closely follow the diagonal reference line, suggesting that the distribution of the Duke Activity Status Index approximates normality. Based on DASI [16], a validated tool for assessing physical functioning.

Figure 2 is a typical Q-Q plot illustrating the variation in Duke Activity Status Index (DASI) scores. The data provides a plot that indicates good agreement between the observed data points and the normal distribution we expected. The distribution of the DASI scores, as shown in the data points, is approximately normal, with the closest fit to the reference diagonal line. This implies that the questionnaire-measured variable has a normal distribution, which is typically an assumption in many statistical tests.

Table 4 shows the intercorrelation between the Vitamin D Deficiency Risk Assessment Questionnaire and Duke Activity Status Index performed using Pearson correlation. The two variables revealed a significant negative correlation (r = -0.271, t = -5.63, p < 0.001), which is a sign that the higher the response to vitamin D deficiency risk status, the lower was the report of physical functioning in individuals. This implies that lower vitamin D is correlated with a lower level of physical activity or the capacity to exercise.

**Table 4:** Intercorrelation Between Study Variables Note. Based on VDRAQ [17], which assesses vitamin D deficiency risk based on lifestyle and demographic factors, and DASI [16], a validated tool for assessing physical functioning. VDRAQ was developed by Alzahrani and Asghar (2025) to assess the risk of vitamin D deficiency based on factors such as sun exposure, BMI, physical activity, and diet. Reference [17] refers to this validated tool. t = test statistics; **p < 0.001 considered significant; correlation = Pearson Correlation

Variable	VDRAQ	DASI	t	p
Vitamin D Deficiency Risk Assessment Questionnaire	-	-0.271	-	-
Duke Activity Status Index	-0.271	-	-5.63	<0.001**

Additionally, subclinical cardiovascular symptoms, such as fatigue, palpitations, and breathlessness, assessed via the VDRAQ, were found to correlate with DASI scores, further supporting the relationship between vitamin D deficiency and impaired physical functioning. These symptoms were included as part of the VDRAQ, which was used to evaluate the risk of vitamin D deficiency and its impact on physical activity.

Table 5 shows the comparison of the average of the Vitamin D Deficiency Risk Assessment Questionnaire and Duke Activity Status Index between females (n = 203) and males (n = 199). Although differences were statistically significant for both variables (VDRAQ, DASI), post hoc tests were not performed due to the straightforward comparison between the two groups. On the Vitamin D Deficiency Risk Assessment Questionnaire, there was a slightly higher mean score among females (M = 28.58, SD = 4.03) than in males (M = 27.86, SD = 3.74) (t = -1.860, p = 0.050, 95 % CI: -1.483 to 0.041), with a small effect size (Cohen's d = -0.19). Men, on the contrary, scored more on the Duke Activity Status Index (M = 17.15, SD = 1.89) than women (M = 16.80, SD = 1.82), and the differences were found to be statistically significant (t = 1.907, p = 0.040, 95% CI: -0.011 to 0.718), and the effect size was small (Cohen s = 0.19). These results indicate that the risks of vitamin D deficiency might be slightly higher among females, and that males have slightly higher rates of better performance in physical activity.

**Table 5:** Comparison among Variables (Gender) Note. Based on VDRAQ [17], which assesses vitamin D deficiency risk based on lifestyle and demographic factors, and DASI [16], a validated tool for assessing physical functioning. VDRAQ was developed by Alzahrani & Asghar (2025) to assess the risk of vitamin D deficiency based on factors such as sun exposure, BMI, physical activity, and diet. Reference [17] refers to this validated tool. M = mean, SD = standard deviation, LL = lower limit, UL = upper limit; CI = confidence interval; Statistical test = Independent t-test; *p < 0.05 considered significant

Variable	Male (N=203); M±S.D	Female (N=199); M±S.D	t	p	Cl 95% LL	UL	Cohen’s D
Vitamin D Deficiency Risk Assessment Questionnaire	27.86±3.74	28.58±4.03	-1.860	0.05^*^	-1.483	0.041	-0.19
Duke Activity Status Index	17.15±1.89	16.80±1.82	1.907	0.04^*^	-0.011	0.718	0.19

Table 6 presents a comparison of study variables among the three age groups using one-way ANOVA. In the case of Vitamin D Deficiency Risk Assessment Questionnaire, the higher the age group, the higher the score: the lowest score was recorded in a group aged 18-20 years old (M = 27.02, SD = 4.36), followed by a group aged 21-23 years old (M = 27.88, SD = 3.96), and then the highest score was recorded by a group aged 24-26 years old (M = 28.72, SD = 3.6). This variability was significant (F(2, 399) = 4.299, p = 0.014), but of a small effect size (η² = 0.021). In the context of the Duke Activity Status Index, the highest ages in physical activity scores (M = 17.41, SD = 1.83) were observed in the age category of 18-20 years of age, followed by 21-23 years (M = 17.27, SD = 1.99), and the lowest scores were registered in the age group 24-26 years (M = 16.67, SD = 1.71). The difference also proved to be statistically significant (F(2, 399) = 6.010, p = 0.003), with a small-to-moderate effect size (η² = 0.029). These results suggest that the risk of vitamin D deficiency is likely to increase slightly with age, whereas physical activity levels are likely to decrease.

**Table 6:** Comparison of Variables (Age) Note. Based on VDRAQ [17], which assesses vitamin D deficiency risk based on lifestyle and demographic factors, and DASI [16], a validated tool for assessing physical functioning. VDRAQ was developed by Alzahrani & Asghar (2025) to assess the risk of vitamin D deficiency based on factors such as sun exposure, BMI, physical activity, and diet. Reference [17] refers to this validated tool. M = mean, SD = standard deviation, F = ratio of variance between groups to within groups, η² = effect size; Statistical test = One-way ANOVA; *p < 0.05 considered significant; **p < 0.01 considered highly significant.

Variable	18-20 years (N=41); M±SD	21-23 years (N=158); M±SD	24-26 years (N=203); M±SD	p	F (2,399)	η2
Vitamin D Deficiency Risk Assessment Questionnaire	27.02±4.36	27.88±3.96	28.72±3.68	0.014^*^	4.299	0.021
Duke Activity Status Index	17.41±1.83	17.27±1.99	16.67±1.71	0.003^**^	6.010	0.029

While the ANOVA results were significant, post hoc tests were not performed because the analysis focused on examining group differences directly via ANOVA. Future studies could benefit from post hoc analyses if individual group differences require further exploration.

Table 7 presents a comparison of the Vitamin D risk of deficiency and physical activity across various categories of marital status. There was a marked disparity across the Vitamin D deficiency risk scores domains (F(3, 398) = 11.840, p < 0.001), and the effect size was moderate (η² = 0.082). There has been a higher score of risk in the divorce group (M = 29.08, SD = 3.61) as well as in those who refused to give their marital status (M = 29.06, SD = 3.68), as opposed to the single (M = 27.93, SD = 3.87) and married groups (M = 25.65, SD = 3.87), which denotes the likelihood of Vitamin D deficiency among these populations. Instead, there were no overall differences in physical activity levels, as assessed by the Duke Activity Status Index (F(3,398) = 2.130, p = 0.096), with an effect size of small magnitude (η² = 0.016). It means that marriage can alter the risk of vitamin D deficiency but does not significantly influence the amount of physical activity that is self-reported.

**Table 7:** Comparison of Variables (Marital Status) Note. Note. Based on VDRAQ [17], which assesses vitamin D deficiency risk based on lifestyle and demographic factors, and DASI [16], a validated tool for assessing physical functioning. VDRAQ was developed by Alzahrani and Asghar (2025) to assess the risk of vitamin D deficiency based on factors such as sun exposure, BMI, physical activity, and diet. Reference [17] refers to this validated tool. M = mean, SD = standard deviation, F = ratio of variance between groups to within groups, η2 = effect size; Statistical test = One-way ANOVA; **p<0.01 considered significant

Variable	Single (N=147); M±SD	Married (N=51); M±SD	Divorced (N=150); M±SD	Prefer not to say (N=54); M±SD	p	F (3,398)	η2
Vitamin D Deficiency Risk Assessment Questionnaire	27.93±3.87	25.65±3.87	29.08±3.61	29.06±3.68	<0.001^**^	11.840	0.082
Duke Activity Status Index	17.27±1.96	17.02±1.56	16.77±1.91	16.72±1.69	0.096	2.130	0.016

Participants who selected ‘prefer not to say’ for marital status were included in the analysis as a separate category to account for potential bias. However, this category was excluded in some inferential analyses where it was not relevant or interpretable.

Table 8 presents the outcomes of a linear regression analysis to investigate the correlation between scores on the VDRAQ and functional capacity, as measured by the DASI. The results demonstrated a significant negative correlation between VDRAQ scores and DASI scores (B = -0.130, p < 0.001), indicating that a higher risk of vitamin D deficiency is associated with lower functional capacity scores. The 95% confidence interval for the unstandardized coefficient ranged from -0.175 to -0.084, confirming the precision of the estimate. The model explained approximately 7.3% of the variance in DASI scores (R² = 0.073; Adjusted R² = 0.071), suggesting a modest but meaningful relationship.

**Table 8:** Linear Regression Analysis Predicting Duke Activity Status Index (DASI) Scores using Vitamin D Deficiency Risk Assessment Questionnaire (VDRAQ) Note. Based on VDRAQ [17], which assesses vitamin D deficiency risk based on lifestyle and demographic factors, and DASI [16], a validated tool for assessing physical functioning. VDRAQ was developed by Alzahrani and Asghar (2025) to assess the risk of vitamin D deficiency based on factors such as sun exposure, BMI, physical activity, and diet. Reference [17] refers to this validated tool. B = coefficient, SE = standard error, β = standardized coefficient, LL = Lower limit, UL = Upper limit; Cl = confidence interval, **p<0.01 considered significant

Variable	B	95% Cl LL	UL	SE	β	P
Constant	20.633	19.343	21.923	0.656	-	<0.001^**^
Vitamin D Deficiency Risk Assessment Questionnaire	-0.130	-0.175	-0.084	0.023	-0.271	<0.001^**^
Model R²	0.073	-	-	-	-	-
Adjusted R²	0.071	-	-	-	-	-

Table 9 presents the distribution of participants by age, marital status, and employment status. The data indicate a correlation between age and marital status (χ² = 29.6, df = 6, p < 0.001), as the majority of respondents in the 18-20 age group were single. In contrast, the most permanent marital status of respondents in the 24-26 age group was either married or divorced. The relationship between age and employment status was not significant, however(χ²(10) = 16.6, p = 0.083), so there was no significant difference in employment types based on age group. Far and wide, the most widely utilized groups were full-time students and full-time workers, with aging participants more likely to be fully employed.

**Table 9:** Descriptive Statistics of Demographic Variables (Age, Marital Status, Employment Status) f = frequency; % = percentage; df = degrees of freedom; p = level of significance; p-values calculated using the chi-square test; *p < 0.05 considered significant; **p < 0.01 considered highly significant.

Variables	f	Single	Married	Divorced	Marital Status: I prefer not to say	df	p	x^2^	Full-time student	Part-time student + part-time work	Employed full-time	Unemployed	Intern/trainee	Freelancing/self-employed	df	p	x^2^
Age	-	-	-	-	-	6	<0.001^**^	29.6	-	-	-	-	-	-	10	0.083	16.6
18-20 years	41	18	11	9	3	-	-	-	3	18	9	8	2	1	-	-	-
21-23 years	158	72	20	53	13	-	-	-	12	40	60	22	15	9	-	-	-
24-26 years	203	57	20	88	38	-	-	-	12	41	68	41	25	16	-	-	-

## Discussion

This research examined the association between the deficiency of vitamin D and subclinical cardiovascular symptoms in young adults in Pakistan. The results of our study revealed a substantial inverse correlation between the risk of vitamin D deficiency and physical functioning. The same findings have been reported in past studies where people with low levels of vitamin D had lower physical activity and worse self-reported health [18]. Subclinical cardiovascular symptoms (such as fatigue and breathlessness) were self-reported via the VDRAQ, while DASI measured physical functioning. Although both are related to vitamin D deficiency, they assess different aspects of health, with VDRAQ focusing on symptoms and DASI on functional capacity.

In our research, we discovered a marginally increased likelihood of vitamin D deficiency in females. This is, however, in contrast with another population-based study (conducted among Saudi adults aged 30-75 years across 18 primary healthcare centers in Riyadh), which recorded greater actual levels of deficiency in males. Such disparities could be caused by the way the assessments were given, population differences, or environmental and lifestyle changes [19]. Our findings revealed that males were comparatively more physically active as compared to females. The finding is in line with the previous studies, which have reported that gender disparities in physical activities start early and elevate over time as a result of both biological and non-biological factors [20].

The results of our study showed that the risk of vitamin D deficiency was lower in younger respondents (18-20 years old) and gradually increased with age. Nonetheless, another study found a higher prevalence of deficiency in younger adults (30-50 years) compared to older individuals and suggested the risk pattern may shift with age and population [19]. The patterns of vitamin D deficiency among age groups in the observed trends can also indicate cultural and contextual dependencies peculiar to Pakistani youths, including exposure to the sun, nutrition, and social prescriptions. Lifestyle factors in younger age groups may vary and determine the level of vitamin D [7]. Our analysis revealed a decrease in physical activity with increasing age, as members aged between 24 and 26 were reported as less active compared to their counterparts aged 18 to 20. Other studies have shown similar results, indicating less physical activity in early adulthood [21].

In our study, married participants were found to be at a much-reduced risk of developing vitamin D deficiency in comparison to that experienced by single, divorced, or undisclosed participants. It is consistent with the existing evidence that lower vitamin D3 and calcium concentrations were observed among unmarried females, indicating that marital status can change the vitamin D status, potentially due to lifestyle or dietary variations [22]. However, while we found an association between marital status and vitamin D deficiency, this relationship is observational, and we cannot claim causality. Other underlying factors, such as lifestyle habits, dietary patterns, or social support, may also contribute to these outcomes. In our research, we discovered that single participants were more physically active than the other marital groups, but this difference was not significant. Earlier studies, however, have found that married people incur more total energy, perhaps because they get more engaged in customary and domestic physical activities [23]. The high proportion of divorced participants in this study may not accurately represent the general young adult population in Pakistan. This could be a result of the specific recruitment settings, where divorced individuals were more likely to participate. Therefore, the marital status variable should be considered with caution when interpreting trends in vitamin D deficiency. The overrepresentation of this group might have influenced the observed trends, and further research should aim to include a more balanced representation of marital status to better reflect the general population.

In our study, we discovered a significant association between a higher risk of vitamin D deficiency and lower physical activity. This agrees with the earlier stated research in dialysis patients, where lower serum vitamin D levels have been attributed to lower levels of physical activity and worsened mental health outcomes [18]. While dialysis patients have distinct health profiles compared to the general population, the association between vitamin D deficiency and physical functioning observed in this population provides valuable insights that may be relevant to understanding similar patterns in other groups. However, it is important to note that, this is an observational association, and the relationship between vitamin D deficiency and physical activity does not imply causality. Other underlying factors may also contribute to these outcomes. Additionally, the small effect size suggests that while vitamin D deficiency is associated with lower physical functioning, its real-world impact may be limited, and other factors may also play a significant role.

In our results, there was a strong relationship between age and marital status, where younger people were mainly single and had high divorce rates in the older age groups. They are congruent with existing studies, which have reported age differences in marital status and related constructs of psychological hope, especially low levels of hope among older, divorced, or widowed people [24]. We found that employment status was different across the age groups, with the younger age groups more likely to be students or part-time workers and the older age groups involved in full-time or flexible work. This is consistent with previous research findings, which highlighted the role of age in shaping employment processes and workforce participation [25].

Limitations and future directions

There are a few limitations in this research that must be mentioned. To start with, a cross-sectional study does not allow for determining the cause-and-effect relationship between vitamin deficiency and cardiovascular symptoms. Whether low vitamin D is a cause of low cardiovascular fitness or a sedentary lifestyle - which reduces sun exposure - that causes deficiency, is not clear. Secondly, both vitamin D risk and physical activity were measured using self-reported questionnaires, which can create biases in responses and do not have equivalent outcomes to clinical diagnoses or laboratory evaluations, including the level of serum 25(OH)D. Thirdly, convenience sampling restricts the scope of the results to the community and educational group because persons who want to take part in this study will not reflect the overall population.

Another limitation is that the Vitamin D Deficiency Risk Assessment Questionnaire (VDRAQ) used in this study has not undergone specific reliability and validity testing for the current population. Although it has demonstrated high performance in prior studies, further testing is necessary to confirm its consistency and applicability in this context.

To establish a cause-and-effect relationship and identify the outcome of the associated modifications in cardiovascular operation after vitamin D supplementation, future studies should be longitudinal or interventional. Biochemical determination of the serum vitamin D levels should be incorporated to give stronger diagnostic accuracy and allow stratification by degrees of deficiency. The external validity can be enhanced by not only conducting a study in more regions but also including a more diverse set of individuals. A higher-than-expected percentage of participants reported being divorced, which may not accurately reflect the broader young adult population in Pakistan. This could limit the generalizability of the findings. Future studies should aim for a more diverse and representative sample to improve the external validity of the results. It should be mentioned that the Duke Activity Status Index (DASI) measures mainly functional capacity but not cardiovascular symptoms directly. Although it brings important insights into the physical functioning, it might not be sufficient to help us understand the extent of subclinical cardiovascular problems, and it is possible to keep this shortcoming in mind when assessing the results.

Other psychosocial mediators like diet, mental health, and social support can also prove more revealing when it comes to the intricate nature of the relationship between vitamin D status and cardiovascular health in young adults. A potential limitation of this study is the lack of control for confounding factors, such as sun exposure duration, dietary intake, and other environmental factors, which may influence vitamin D levels and cardiovascular health. Future studies should aim to incorporate these factors to isolate the effects of vitamin D deficiency better. Given the observed gender differences in vitamin D deficiency and physical activity, future studies should incorporate sex-stratified biochemical data to better understand the biological mechanisms underlying these differences.

## Conclusions

In conclusion, the study has reported a correlation* *between the risk of vitamin D deficiency and poor physical performance, indicating the prospect of premature subclinical cardiovascular manifestations in Pakistani youths. However, it is essential to note that this relationship is correlational rather than causal due to the study's cross-sectional design. These results are essential to dispel the traditional belief that the young population is excluded from cardiovascular risk and to promote aggressive screening and lifestyle measures. Poor physical performance was assessed using DASI scores, which measure physical capacity rather than self-reported cardiovascular symptoms, such as fatigue or palpitations. The results emphasize the necessity of informing and enhancing the quality of the diet, as well as reducing the harm caused by exposure to sunlight. This includes the need to maintain a balance between sufficient sun exposure to promote vitamin D production and adequate sun protection. Environmental methods, such as school-based programs and urban design, can facilitate outdoor activities that promote cardiovascular health among young adults through vitamin D-increasing activities. Since vitamin D deficiency is a preventable disease, the prevention of its effects by identifying and intervening at an early age may become a cost-effective measure in preventive cardiology.
